# Regulatory rare variants of the dopaminergic gene *ANKK1* as potential risk factors for Parkinson’s disease

**DOI:** 10.1038/s41598-021-89300-6

**Published:** 2021-05-10

**Authors:** Estela Pérez-Santamarina, Pedro García-Ruiz, Dolores Martínez-Rubio, Mario Ezquerra, Irene Pla-Navarro, Jorge Puente, María José Martí, Francesc Palau, Janet Hoenicka

**Affiliations:** 1 Centro de Investigación Biomédica en Red de Enfermedades Raras (CIBERER), ISCIII, Madrid, Spain; 2 Centro de Investigación Príncipe Felipe (CIPF), Valencia, Spain; 3 Unit of Movement Disorders, Department of Neurology, Fundación Jimenez Díaz, Madrid, Spain; 4 Laboratory of Neurodegenerative Disorders, Department of Neurology, Hospital Clínic of Barcelona, IDIBAPS, Barcelona, Spain; 5 LabGenetics, Madrid, Spain; 6 Movement Disorders Unit, Department of Neurology, Hospital Clínic of Barcelona, IDIBAPS, Barcelona, Spain; 7 Laboratory of Neurogenetics and Molecular Medicine, Neurogenetics and Molecular Medicine Research Group, Institut de Recerca Sant Joan de Déu, C/ Santa Rosa 39-57, Esplugues de Llobrega, 08950 tBarcelona, Spain; 8 Department of Genetic Medicine, Hospital Sant Joan de Déu, Barcelona, Spain; 9 ICMID, Hospital Clínic, and Division of Pediatrics, University of Barcelona School of Medicine and Health Sciences, Barcelona, Spain; 10 Present Address: University of East Anglia, Norwich, UK; 11 Present Address: Unit of Rare Neurodegenerative Diseases, CIPF, Valencia, Spain; 12 Present Address: Rare Diseases Joint Units, CIPF-IIS La Fe and INCLIVA, Valencia, Spain

**Keywords:** Neurological disorders, Parkinson's disease, Genetics, Diseases, Risk factors

## Abstract

Parkinson’s disease (PD) is characterized by cerebral dopamine depletion that causes motor and cognitive deficits. The dopamine-related gene *ANKK1* has been associated with neuropsychiatric disorders with a dopaminergic deficiency in the striatum. This study aims to define the contribution of *ANKK1* rare variants in PD. We found in 10 out of 535 PD patients 6 *ANKK1* heterozygous rare alleles located at the 5′UTR, the first exon, intron 1, and the nearby enhancer located 2.6 kb upstream. All 6 *ANKK1* single nucleotide variants were located in conserved regulatory regions and showed significant allele-dependent effects on gene regulation in vitro*. ANKK1* variant carriers did not show other PD-causing Mendelian mutations. Nevertheless, four patients were heterozygous carriers of rare variants of *ATP7B* gene, which is related to catecholamines. We also found an association between the polymorphic rs7107223 of the *ANKK1* enhancer and PD in two independent clinical series (*P* = 0.007 and 0.021). rs7107223 functional analysis showed significant allele-dependent effects on both gene regulation and dopaminergic response. In conclusion, we have identified in PD patients functional variants at the *ANKK1* locus highlighting the possible relevance of rare variants and non-coding regulatory regions in both the genetics of PD and the dopaminergic vulnerability of this disease.

## Introduction

Parkinson’s disease (PD) is a neurodegenerative disorder characterized by progressive movement disability and a variety of non-motor symptoms. PD patients show tremor, rigidity, bradykinesia, and postural instability caused by the selective death of dopaminergic neurons in the *substantia nigra*^1^. The dopamine deficiency in PD also impairs a wide network of brain areas that cause cognitive deficits. In addition, other neurotransmitters are also implicated including the cholinergic system, which partially explains cognitive deficits in advanced stages^2^. Genetic studies of familial and sporadic PD patients have identified more than 27 PD-associated genes^3,4^. However, there is considerable missing heritability and therefore the need to identify novel genes.

Single nucleotide variations (SNVs) of the Ankyrin repeat and kinase domain containing I gene (*ANKK1*; MIM*608774) have been reported to be associated with neuro-psychiatry disorders characterized by a dopaminergic deficiency in the striatum^5^. In particular, the *Taq*IA SNV (rs18000497) has been previously linked to alcoholism, antisocial traits^6^, and other psychiatric disorders^5^. *Taq*IA has also been associated with both PD vulnerability^7,8^ and clinical features of PD patients such as dopamine agonist response^9^ or impulse control disorders as a side effect of pharmacological treatment^10,11^.

The *ANKK1* gene and protein have been related to the dopaminergic system^12–14^. In silico analysis of the A*NKK1 locus* identified share haplotypic blocks with the neighbor gene *DRD2* (Dopamine Receptor D2 gene)^15^ and it has been proposed that *ANKK1*/*DRD2 locus* is a genomic substrate for cognitive traits^16^. *Taq*IA *ANKK1* SNV is in linkage disequilibrium with *DRD2* variants that regulate the expression of the long and the short isoforms of the DRD2^17^ and its distribution in the ventral striatum^18^. The C957T *DRD2* SNV (rs6277) is a marker of a regulatory sequence at the 5′ UTR of the *ANKK1* gene^16^. Additionally, an association between *Taq*IA and striatal activity of the aromatic L-amino acid decarboxylase has been described^19^. Therefore, there is evidence that variations of the *ANKK1* gene have a significant impact on the functioning of the dopaminergic system in the brain.

Given that cerebral dopamine depletion is a hallmark of PD here we evaluate in PD patients the eight exons and 5′ regulatory regions of *ANKK1* as a new PD risk candidate gene. We identified PD-related regulatory SNVs in the *ANKK1 locus*.

## Results

### PD-related *ANKK1* rare variants affect gene expression

**Figure 1:**
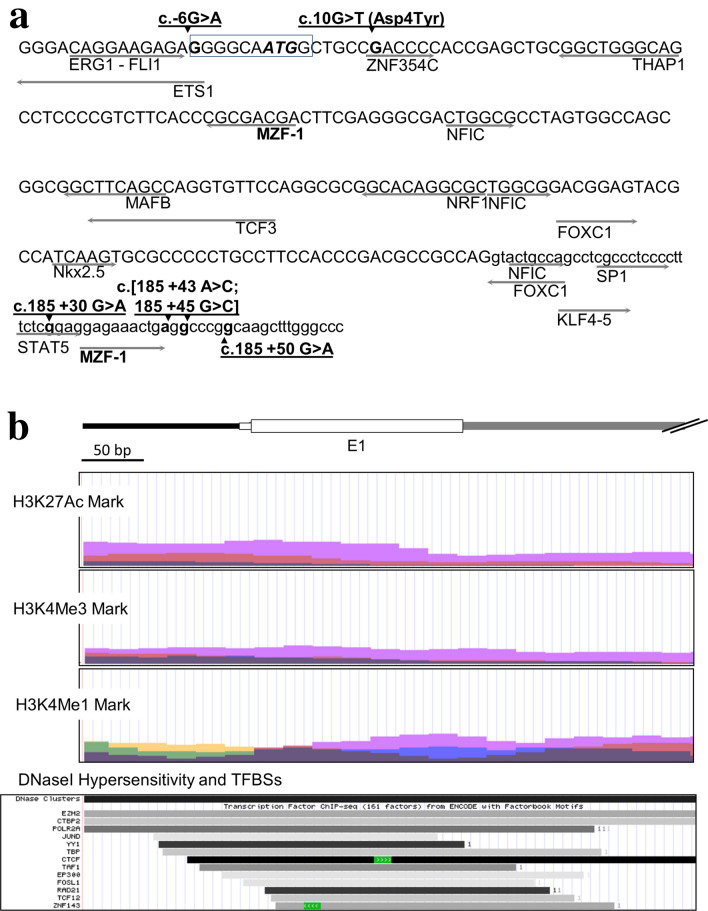
*ANKK1* SNVs related to PD are located in the conserved 5′ regulatory regions of the gene. (**a**) Sequence of *ANKK1* Exon 1 (capital letters) and intron 1 (lowercase) annotated with rare PD-related SNVs (bold), Kozak sequence (square), and transcription factor binding sites (TFBSs) identified by JASPAR (grey arrows). (**b**) ENCODE chromatin modifications marks of *ANKK1* exon 1/intron 1 region in Human Skeletal Muscle Myoblasts (HSMM, green), Human Umbilical Vein Endothelial Cells (HUVEC, blue), Normal human epidermal keratinocytes (NHEK, violet) cell lines. Histone acetylation and methylation marks for H3K27Ac, H3K4m3 and H3K4m1 indicate promoters and enhancers. DNaseI hypersensitivity indicates regulatory regions and TFBSs.

Firstly, we studied variation at the eight exons of *ANKK1* from 71 PD patients of the FJD series using DHPLC and Sanger sequencing. We found three heterozygous SNVs in two patients. PD-03 carried the coding variant c.10G>T located at exon 1 (rs35657708, p.Asp4Tyr) while PD-01 had the variants c.185 + 43A>C (rs769430211) and c.185 + 45G>C (rs780054479) at intron 1 (Fig. 1a). The cloning and sequencing of PD-01 intron 1 showed that the two variants were in *cis* (Fig. S1g). The screening of c.10G>T and c.[185 + 43A>C;185 + 45G>C] by DHPLC in samples from the DNBCIII series revealed they were absent in 288 ethnic-matched healthy controls and 200 PD patients. We found in one PD patient from DNBCIII the c.185 + 30G>A SNV (rs545702309) (Fig. 1a). After, we screened these variants in the independent HC PD series of 264 patients. Two samples (PD-07 and PD-08) were heterozygous for c.10G>T and PD-06 carried the allele c.[185 + 43A>C;185 + 45G>C]. In addition, we found the novel rare variant c.-6G>A in PD-04 and the rare variant c.185 + 50 G>A in PD-05 (Fig. 1a). All *ANKK1* variants were absent in 288 ethnic-matched healthy controls.

The in silico study of *ANKK1* SNVs using CADD showed that c.10G>T, c.185 + 43A>C and c.185 + 45G>C, found in the two clinical series, have the highest C-scores thus suggesting a higher likelihood of deleterious effect on *ANKK1* gene regulation (Table S1). We also found these variants occur in well-conserved nucleotides among primates (Fig. S2). The transcription factor binding sites (TFBSs) prediction tool JASPAR, and the Encyclopedia of DNA Elements (ENCODE) data, showed that all variants disrupt TFBSs leading to loss or gain of multiple TFBSs (Fig. 1, Table S2). Further analysis of this region using the ENCODE data showed epigenetic marks associated with promoter activity including accessible chromatin and TFBSs (Fig. 1b).

**Figure 2:**
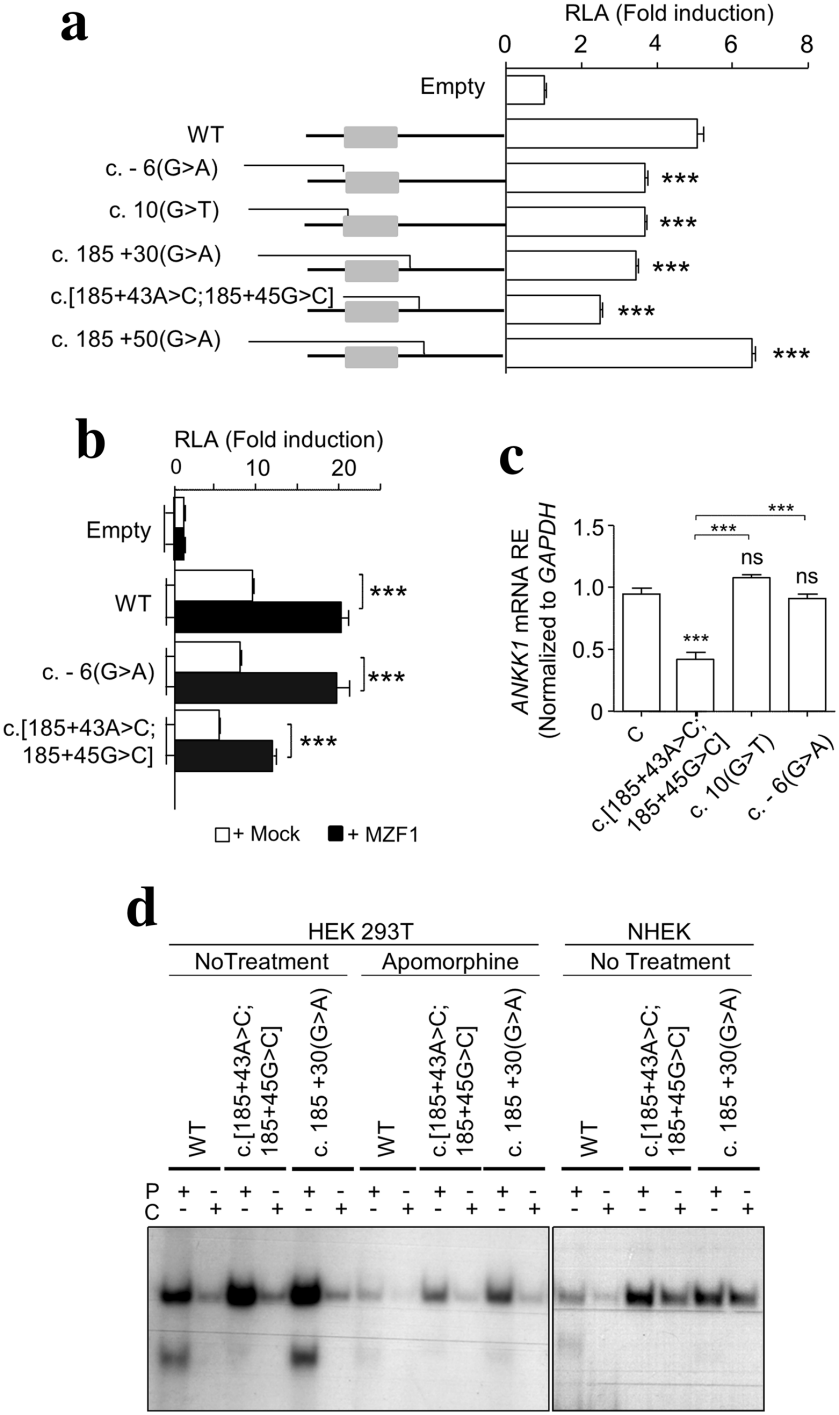
PD-related rare *ANKK1* SNVs showed significant allele-dependent effects on gene regulation. (**a**) Relative luciferase activity (RLA) of WT and rare variants of *ANKK1* exon 1/intron 1 (sequence − 327 up to + 774) cloned in the pGL3-basic vector and transfected in HEK293T cells. (**b**) RLA of *ANKK1* allelic constructs and MZF-1-GFP co-transfection in HEK293T cells. pEGFP-N1 was used as a mock. Data are shown as mean ± SEM (N = 4). (**c**) Real-time RT-PCR analysis of *ANKK1* mRNA expression in whole venous blood from some PD patients carriers of *ANKK1* SNVs and two controls pooled together to make a single sample (N = 3). (**d**) EMSA of WT and *ANKK1* variants using radiolabeled probes (P) and molar fold excess of non-labeled competitors (C) as a control. The assay was carried out in HEK293T (untreated and treated with apomorphine) and NHEK cells. Asterisks indicated statistical significance (*P < 0.05, **P < 0.01, ***P < 0.001), one-way ANOVA followed by Tukey correction was conducted.

Next, we performed a luciferase reporter assay (LRA) to validate the functional consequences of the PD-related *ANKK1* rare variants. For each variant and WT, luciferase reporter constructs were generated and transfected into HEK293T cells to determine the transcriptional activity. All variants showed significant allele-dependent effects on gene regulation when compared to WT. c.-6G > A, c.10G>T, c.185 + 30G>A and c.[185 + 43A>C;185 + 45G>C] drove lower expression upon reporter activity while the opposite effect was observed for c.185 + 50 G>A (Fig. 2a). Given that c.[185 + 43A>C;185 + 45G>C] showed the lowest expression levels and is immediately adjacent to the binding site of the transcription factor MZF-1, we studied the impact of this variant upon MZF-1 binding. We found a significant increase of luciferase activity in cells co-transfected with *ANKK1* alleles constructs and plasmid MZF-1-GFP. We found that WT and c.-6G>A alleles showed similar expression levels while c.[185 + 43A>C; 185 + 45G>C] was still significantly lower (Fig. 2b) thus confirming the negative effect of this variant upon *ANKK1* transcription activity.

We next measured *ANKK1* RNA levels in a sample of peripheral blood cells from PD patients that carry c.-6G>A, c.10G>T, or c.[185 + 43A>C;185 + 45G>C]. In contrast with LRA results, only the patient carrying the c.[185 + 43A>C;185 + 45G>C] allele was different from the WT which suggests that the effect of *ANKK1* could be tissue-specific (Fig. 2c).

### *ANKK1* variants were differentially affected upon dopaminergic treatment

Since the expression of *ANKK1* is closely related to the functioning of the dopaminergic system^13^, we also studied the effect of apomorphine (dopaminergic agonist) treatment upon *ANKK1* variants. In LRA using apomorphine no effect was found upon expression of WT, c.-6G>A, c.10G>T, and c. [185 + 43A>C; 185 + 45G>C] alleles. In contrast, c.185 + 30G>A showed a significant upregulation while we observed in c.185 + 50G>A the opposite effect decreasing the expression up to WT levels (Fig. S3). To further study apomorphine impact upon *ANKK1* variants, an electrophoretic mobility shift assay (EMSA) was performed. In untreated cells, two DNA–protein complexes appeared as a band shift pattern in WT and c.185 + 30G>A while c. [185 + 43A>C; 185 + 45G>C] showed a single band shift (Fig. 2d, Fig. S4a). Apomorphine treatment caused a strikingly decreased binding. EMSA using Primary Normal Human Epidermal Keratinocytes (NHEK) nuclear proteins confirmed quantitative and qualitative differences between the WT and the variants c.185 + 30G>A and c.[185 + 43A>C; 185 + 45G>C] (Fig. 2d, Figure S4b).

### rs7107223 SNV located at *ANKK1* enhancer is associated with PD

**Figure 3:**
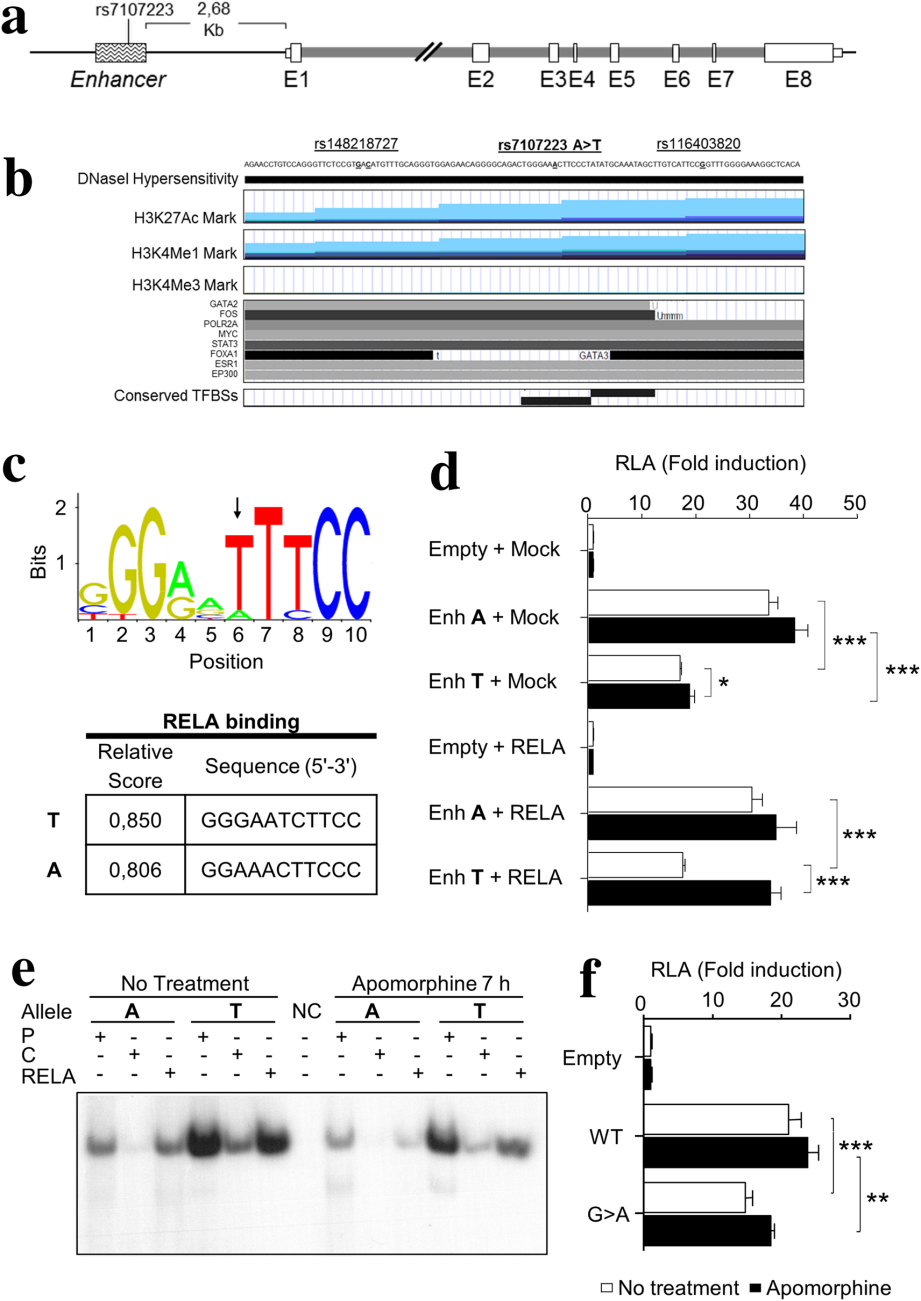
*ANKK1* enhancer activity is affected by rs7107223 alleles. (**a**) *ANKK1 locus* showing both an enhancer located 2.68 Kb upstream (wave pattern-block) and coding exons (white blocks). (**b**) ENCODE chromatin modifications marks of *ANKK1* enhancer in HUVEC (blue), NHEK (violet), and HSMM (green) cell lines. Histone acetylation and methylation marks for H3K27Ac, H3K4m3, and H3K4m1 indicate an enhancer. DNaseI hypersensitivity indicates regulatory regions and TFBSs (**c**) Human binding motif of RELA and relative scores and sequence for RELA BS in the rs7107223 region. Data obtained from JASPAR database. (**d**) RLA of rs7107223 alleles constructs or after co-transfection with T7-RelA plasmid. (**e**) EMSA of rs7107223 alleles using radiolabeled probes (P), molar fold excess of non-labeled competitors (C) as a control and RELA as a competitor. (**f**) RLA of g.113384813G>A alleles constructs. HEK293T cells untreated and treated with apomorphine were used in D, E and F. Data are shown as mean ± SEM (N = 4). Asterisks indicated statistical significance (*P < 0.05, **P < 0.01, ***P < 0.001), one-way ANOVA followed by Tukey correction was conducted.

Since the *ANKK1* study in PD patients identified rare SNVs that affect transcriptional activity, we hypothesized that other nearby regulatory genomic elements could also be involved in PD vulnerability. ENCODE data showed a regulatory element at 2.6 Kb from the *ANKK1* ATG start codon (Fig. 3a, Fig. S5a). This region shows chromatin modification marks that indicate enhancers and conserved elements in primates (6 elements) and *M. musculus* (248-bp region) (http://ecrbrowser.dcode.org/) (Fig. S5b). Further analysis using Ensemble and Haploview software detected polymorphic SNVs in this enhancer, among them, the rs7107223 were selected for further study. This SNV is located at a conserved TFBSs region (Fig. 3b, Fig. S5) and JASPAR software predicted that the A-to-T allele change causes the loss of FOXO3 and FEV binding sites (BSs) and the gain of an E2F6 BS.

rs7107223 was genotyped in a population series of 200 PD patients and 288 control individuals from a DNBCIII to perform an association study. The rs7107223 met the Hardy–Weinberg’s equilibrium in both populations (χ^2^ = 0.69, *P* = 0.4067 for the patients; χ^2^ = 0.86, *P* = 0.3551 for the controls). In the patients' sample, a significant increase of the A allele was observed when compared with controls (A Allele: 48.73 *vs* 37.85%; χ^2^ = 9.55, *P* = 0.0008). This increment is produced at the expense of the T/T homozygous genotype thus causing the significant increase of A/A homozygous genotype under the log-additive model (Table 1). This positive association was replicated in an independent sample of the 264 PD patients from HC (Table 1).

**Table 1:** Genotypic association between rs710223 and PD in two independent clinical series.

Sample	PD Freq			Control Freq			O.R. (95% C.I.)	Model	Genotypic *P* value
	AA	AT	TT	AA	AT	TT			
1	0.23	0.515	0.255	0.142	0.472	0.385	1.57 (1.21–2.05)	Log-additive	< 0.0007
2	0.187	0.519	0.293	0.142	0.472	0.385	1.31 (1.04–1.85)	Log-additive	0.021

Genotypic test calculated in SNPstats software with sex- and age-adjustment of *P* values. Sample 1 from DNA Bank: PD: 200, Controls: 268; Sample 2 a clinical series of 264 PD patients. The genotypic test was chosen as the genetic test model with a lower AIC/lower *P* value. Freq. = frequency; O.R. = Odds Ratio.

To investigate the functional consequences of rs7107223 alleles, we performed both LRA and EMSA studies. LRA showed a strong promoter activity for the two rs7107223 alleles although the A allele drove 3.5-fold more expression upon reporter activity (A allele Mean (M) = 43.76 ± 1.016 *vs* T allele M = 12.35 ± 0.5395; *P* < 00.0001). As rs7107223 affects the BS of RELA that is part of the NF-kappa-B complex (Fig. 3c), we also performed an LRA after co-transfection with the T7-RelA plasmid (Fig. 3d). No effect was observed with overexpression of RELA. However, in cells treated with apomorphine, we observed the increment of the rs7107223 T allele in either absence (*P* < 0.05) or presence of RELA (*P* < 0.0001) (Fig. 3d) while the A allele showed no changes after treatment. In contrast, EMSA studies showed in both alleles a competition RELA effect in apomorphine-treated samples thus indicating the bindings of this TF regardless of genotype (Fig. 3e, Fig. S6).

To further study this enhancer in PD, a 795-bp region around the rs7107223 SNV was sequenced in 71 FJD and 50 HC PD series. We found the novel SNV g.113384813 G>A in 2 patients. This SNV causes the loss of TFBSs (FoxA1, ARNT::HIF1A, Ahr::Arnt, Atf3, MAFG::NFE2L1, Atf1, MEIS1, MEIS2, MEIS3, and Pax2) and the gain of GMEB2 and GRHL1 BSs. The screening of g.113384813 G>A revealed that it was absent in 288 controls and 414 PD patients. LRA showed a strong promoter activity and no response to apomorphine for g.113384813 G>A alleles. The comparison between the G (WT) and A alleles revealed a significant decrease of activity for the novel allele (*P*˂0.0001) (Fig. 3f).

### Patients carrying *ANKK1* variants do not have mutations in PD-causing Mendelian genes

To better know the genetics underlying PD in patients that carry *ANKK1* rare SNVs, these samples were also analyzed with an NGS custom panel and MLPA. The panel includes 24 PD-related genes and MLPA analysis interrogates deletions or duplications in 8 PD genes and the presence of both A30P in the *SNCA* (α-Synuclein) and G2019S in the *LRRK2* (Leucine-rich repeat kinase 2) genes (Supplementary Methods). None of the patients carried PD-causing Mendelian mutations, nevertheless, we found heterozygous patients for rare variants in *NR4A2* (Nuclear Receptor Subfamily 4 Group A Member 2), in *ATP7B* (ATPase, Cu(2+)-transporting, beta polypeptide), *GBA* (Glucosidase, beta, acid) and *LRRK2* (Table S3). Notably, four patients had rare heterozygous variants in *ATP7B*. PD-08 that shows the earliest age of onset of the disease and cognitive decline, carries a pathogenic variant in *GBA,* a known risk factor for PD^4^. We also found patients with rare variants of *LRRK2* gene.

PD patients who carry *ANKK1* rare SNVs showed significant variability in main clinical symptoms, motor complications, and cognitive decline, with limited phenotype/genotype correlation, and the age of onset was from early to classical onset (Table S7). Altogether and the lack of family history in most of these patients suggest that *ANKK1* rare variants are risk factors of PD vulnerability and not Mendelian cause of disease.

## Discussion

In this work, the study of the *ANKK1 locus* in PD showed that 10 out of 535 patients (1.9%) carried rare variants within regulatory regions. All 6 *ANKK1* SNVs were located in conserved regulatory regions, showed significant allele-dependent effects on gene regulation, and had extremely low frequency or were absent in international or Spanish databases of control populations.

*ANKK1* variants' contribution to the vulnerability of PD should be investigated in independent patients’ series. Despite this limitation, we believe that the finding of PD-related rare variants in regulatory regions deserves greater attention in the future search for genetic risk factors of PD. On the one hand, there is a need to identify and analyze rare genetic variants because they could be moderate risk factors contributing to PD vulnerability. On the other, genome-wide association studies (GWAS) in human diseases have shown that the majority of disease-associated variants lie outside protein-coding regions. In PD, a GWAS meta-analysis reported 11 out of 17 novel *loci* in linkage disequilibrium with variants predicted to affect TFBSs and 3 *loci* of genes that encode TFs^20^. Focusing on the dopaminergic system, the study of the epigenome landscape of PD has shown hypermethylation of regulatory regions associated with downregulation of a network of TF including FOXA1 that is related to dopaminergic neurons^21^. It has been reported that PD is associated with deregulations of a dopamine-modulated gene network in the striatum that is relevant for non-motor and motor symptoms^22^. Our finding of PD-related rare *ANKK1* variants also highlights the possible contribution of primary dopaminergic dysfunction as a condition of PD vulnerability. Moreover, PD patients carrying regulatory *ANKK1* variants do not have disease-causing mutations in PD Mendelian genes. Five patients carry rare variants in *NR4A2* and *ATP7B* genes, which could affect catecholaminergic homeostasis in the brain. The nuclear receptor NURR1 encode by *NR4A2* is expressed in midbrain dopamine neurons during neurodevelopment and in adulthood and, its down-regulation contributes to dopamine neuron dysfunction^23^. The ATPase ATP7B is also related to the dopaminergic pathway in the brain since this protein contributes to catecholamine metabolism^24^. Four patients had rare heterozygous variants in *ATP7B* that could contribute as risk factors in catecholaminergic neuron dysfunction in the early stages of PD. Indeed, it has been reported that single *ATP7B* regulatory SNV may confer susceptibility for late-onset major depression and parkinsonism^25^ as well as *ATP7B* coding mutations in early-onset PD^26^. Our findings in PD patients in *ANKK1* and other catecholaminergic-related genes support the role of genetic variability in the dopaminergic pathway as a risk factor for the disease. On the other hand, 2 patients carry rare variants in the *GBA* or *LRRK2* genes. GBA is one of the most recognized PD modifying genes in sporadic cases^4^, and the ambiguous classification of the *LRRK2* variant does not allow reaching the genetic diagnosis. These results altogether suggest these rare variants, including regulatory *ANKK1* variants, contribute to the PD pathophysiology by increasing PD susceptibility.

In this work, we also found an association between PD and the functional rs7107223 SNV located at the enhancer nearby *the ANKK1* promoter. Our functional studies showed that while RELA binding to this enhancer was genotype-independent, the transcriptional effect of TF binding was observed only for the rs7107223 T allele and it was dependent on dopaminergic stimulation. These findings suggest the link between dopaminergic function and the regulation of this enhancer. Moreover, rs7107223 is in strong linkage disequilibrium with rs11214594, rs17600713, and rs12360992 located at *ANKK1* intron 1^16^. These *ANKK1* polymorphisms are predicted to have a potential functional impact on the gene expression in the human brain (http://featsnp.org/). Therefore rs7107223 not only is a functional SNV associated with PD but also is a marker for regulatory *ANKK1* SNVs at intron 1. rs7107223 is also linked to the rs6277 SNV of the *DRD2* gene^16^. This SNV has a marked impact on the variability in DRD2 binding features^27^. Since PD shows dopamine depletion in neural pathways innervating the striatum, thalamus, and cortex, it is possible that rs7107223 is also a functional marker of DRD2 variability that would facilitate or hinder compensatory changes in the brain and they may be relevant for clinical phenotype variability in PD patients.

In summary, we have identified functional PD-related rare and polymorphic SNVs in the *ANKK1 locus* that act in *cis* regulatory DNA elements of *ANKK1*. The knowledge of *ANKK1* as a new susceptibility gene for PD shows the relevance of both rare variants and non-coding gene regulatory regions in PD genetics and will help to understand pathophysiological aspects of dopaminergic vulnerability in PD.

## Methods

### Study participants

The study included two independent clinical series of Spanish PD patients. 71 patients were recruited from the Neurology Department of the Fundación Jiménez Díaz (FJD)*,* Madrid, Spain (45 males and 26 females with disease onset between the ages of 35 and 77 years, X = 57.31; σ = 10.54), and 264 patients were from the Hospital Clínic (HC), Barcelona, Spain (157 males and 107 females with disease onset between the ages of 16 and 86 years, X = 53.40; σ = 11.91). The inclusion criteria were PD diagnosis according to the London Brain Bank^28^. The study also included 200 PD patients (aged between 41 and 98 years, X = 69.48; σ = 9.51) from the *DNA National Bank Carlos III* (DNBCIII) at the University of Salamanca, Spain and, 288 healthy controls (aged between 55 and 73 years, X = 60.03; σ = 3.33). The control sample is a collection of DNAs from healthy donors (not diagnosed with any relevant disease) and representative of the population residing in Spain.

The study has been approved by the Clinical Research Ethics Committee of “Institut de Recerca Sant Joan de Déu”, Barcelona (PIC-93-16) and “Fundación Jimenez Díaz”, Madrid (PIC76/2015-FJD). All methods were performed in accordance with the relevant guidelines and regulations set forth by the Declaration of Helsinki. Informed written consent was obtained from all participants.

### *ANKK1* mutation screening

Genomic DNA from peripheral blood was extracted following conventional protocols with proteinase K. *ANKK1* genetic variants at 5′-UTR region and exons 1, 3, 4, 6, and 7 were screened by denaturing high-performance liquid chromatography (DHPLC), while exons 2, 5, and 8 (larger and more polymorphic) by Sanger sequencing. The software *Gene Runner* (version 3.05, *Hastings Software*) was employed for primer’s design using *ANKK1* gene reference sequence NM_178510.1. *ANKK1’s* exons and their flanking intronic regions were amplified by PCR at the specific annealing temperature of each pair of primers (Table S5). The 5′-UTR region of *ANKK1* was amplified by touchdown PCR (TD-PCR).

For DHPLC we used the WAVE System (Transgenomic, Nebraska, USA) according to the manufacturer’s instructions. The conditions for DHPLC were established using Transgenomic Navigator software and adjusted by adding positive and negative controls in each sample set. Sanger sequencing was performed on positive amplicons.

### Genotyping of rs7107223

Touch-down (TD)-PCR was performed using the primers rs7107223-F and rs7107223-R (Table S5) and PCR products digested with *TfiI* (New England Biolabs, Massachusetts, USA). After, genotypes were visualized in 1.8% agarose gel in Tris–acetate-EDTA (TAE) 1X containing GelRed (1:10,000, Biotium). The web tool SNPStats (https://www.snpstats.net/start.htm?q=snpstats/start.htm) was used to analyze the association under five genetic models (codominant, dominant, over‐dominant, recessive, and log‐additive) with an adjustment of age^29^.

### Genetic panel and multiplex ligation-dependent probe amplification (MLPA) analysis

Some PD samples were genotyped in a custom AmpliSeq primer panel for 24 genes (Supplementary methods) designed online (Ion Ampliseq Designer v.1.2.8, https://ampliseq.com; Thermo Fisher Scientific). Library construction was performed according to the Ion AmpliSeq Library Kit 2.0 protocol and the Ion OneTouch System protocol (PNMAN0006957, rev. 6.0; Thermo Fisher Scientific) following manufacturer instructions. Sequencing was performed using the Ion Personal Genome Machine_ (PGM) 200 sequencing kit protocol (PN4474246, rev. D; Thermo Fisher Scientific). The sequencing runs were performed using 520 flows for 130 cycles. For data analysis and variant prioritization pipeline, we used the Torrent Suite (TS) software version 4.0. Five main steps were performed: quality control, alignment, variant calling, variant annotation, and filtering.

MLPA was used for some PD samples to identify large deletions or duplications. MLPA kit P051-D1 (MRC-Holland, Amsterdam, The Netherlands) was used according to manufacturer instructions. The P051-D1 set includes *ATP13A2* (exon 2 and 9), *PARK2* (exons 1–12), *PARK7* (exons 1–7), *PINK1* (exons 1–8), *SNCA* (exons 1–6; point mutation A30P), *LRRK2* (point mutation G2019S).

### Constructions of expression plasmids and luciferase reporter assays (LRA)

Oligonucleotide primers containing engineered *Kpn*I sites were designed for PCR-based amplification of genomic DNA from patients to generate the pGL3 plasmids for each variant (Supplementary methods and Table S6). MZF-1-GFP plasmid containing the Myeloid Zinc Finger 1 gene was kindly provided by Dr. Jiawei Zhou and the pEGFP-N1 (Clontech, Mountain view, California, USA) use as a mock. T7-RelA plasmid containing *RELA* (part of the NF-kappa-B complex) gene was a gift from Warner Greene (Addgene plasmid # 21984).

HEK293T cells were grown in DMEM containing 10% (v/v) fetal bovine serum, 1% penicillin–streptomycin, and 2 mM L-glutamine (Lonza, Basel, Switzerland). NHEK cells were kindly provided by Dr. Pilar Sepúlveda Sanchís. Cells were incubated at 37ºC in 5% CO_2._ For LRA, HEK293T cells were transfected with 500 ng of luciferase expression pGL3 plasmids and 40 ng of renilla expression construct (pRL-TK vector, Promega) using FuGene HD reagent (Promega, Madison, Wisconsin, USA). For co-transfection experiments, 500 ng of MZF-1-GFP or T7-RelA plasmid were transfected with 500 ng of pGL3 plasmids and 40 ng of pRL-TK. In some cases, 24 or 48 h post-transfection, cells were treated with the dopamine receptor agonist (R)-(-) apomorphine (APO) (Sigma, Saint Louis, Missouri, USA) at 10 μM in complete medium with 0.5% serum for 8 h. After 48 h, cells were lysed and luciferase and renilla activity were measured using the Dual Luciferase Reporter Assay System (Promega) and the Wallac Victor2 spectrophotometer (Perkin Elmer, Waltham, Massachusetts, U.S.). Each experiment was performed in triplicate at least 6 times. The ratio of luciferase to renilla activity and the fold increase relative to a mock (control luciferase expression vector) were calculated. Data are expressed as the mean ± standard errors of the mean (SEM) and values of *P* < 0.05 were considered significant. Differences were evaluated by one-way ANOVA followed by Tukey’s multiple comparisons *post-hoc* test using GraphPad v6.0 (CA, USA).

### Electrophoretic mobility shift assay (EMSA)

HEK293T (untreated and treated with apomorphine) and NHEK nuclear extracts were prepared using the NE-PER nuclear and cytoplasmic extraction reagents (Pierce). Double-stranded oligonucleotides (Table S7) were labeled with [γ-^32^P] ATP using a T_4_ polynucleotide kinase (Promega, Wisconsin, USA) and purified on a Microspin G-25 column (Roche, Basilea, Switzerland). For EMSA, 5 µg of nuclear extract were incubated with 1 µg of poly deoxyinosine-deoxycytosine and 15 fmol of the ^32^P-labeled probes in a 20 µl binding reaction containing 1X buffer (50% glycerol; 10 mM Tris–HCl, pH 7.6; 500 mM KCl; 10 mM EDTA; and 1 mM dithiothreitol) for 30 min at room temperature. For the specificity control, a molar excess of unlabeled probe over the radiolabeled oligonucleotide was added to the binding reaction.

After, the reaction mixtures were mixed with loading dye and separated on a 6% nondenaturing polyacrylamide gel (29:1 acrylamide-bisacrylamide) and electrophoresed in a 0.25X Tris-boric buffer at 150 V for 1.5 h. The gel was dried and exposed to X-ray film for autoradiography.

### RNA extraction and RT-PCR form peripheral blood

Whole venous blood was collected into PAXgene Blood RNA Tube (PAX tubes; BD Vacutainer, Plymouth, UK) and the RNA was extracted using the PAXgene Blood RNA System Kit (PreAnalytiX, QIAGEN) following the manufacturer's guidelines. PolyT primers were used for the RT reaction using the Maxima First Strand cDNA Synthesis Kit (Fermentas). All quantitative real-time PCR reactions were carried out in triplicate on a 7500 Fast Real Time PCR System (Applied Biosystems, Foster City, California, USA). Gene expression was analyzed using FastStart Universal SYBR Green Master Rox (Roche). Amplification was performed using 200 ng of cDNA per reaction, at 50ºC for 2 min, 95 °C for 10 min, followed by 45 cycles at 95 °C for 15 s, 60 °C for 1 min and 72 °C for 30 s (extension Samples were quantified by the standard-curve method ^30^ and normalized to the *GAPDH* (glyceraldehyde 3-phosphate dehydrogenase) and *PPIA* (Peptidylprolyl Isomerase A (Cyclophilin A)) housekeeping genes. Results were analyzed with SDS 2.1 software (Applied Biosystems). Baseline values of amplification plots were set automatically and threshold values were kept constant to obtain normalized cycle times and linear regression data. The primers used are shown in Table S6. Data are expressed as the mean ± standard errors of the mean (SEM) and values of *P* < 0.05 were considered significant. Differences were evaluated by one-way ANOVA followed by Tukey´s multiple comparisons *post-hoc* test using GraphPad v6.0 (CA, USA).

### In silico studies

Combined Annotation Dependent Depletion (CADD) was used for scoring the deleteriousness of single nucleotide variants (https://cadd.gs.washington.edu/). For the identification of regulatory regions of the *ANKK1* gene, we used data from The Encyclopedia of DNA Elements as a part of the ENCODE consortium (genome build GRCh37/hg19) ^31^. To search for potential transcription factor binding sites (TFBSs) affected by SNVs we use the JASPAR (http://jaspar.binf.ku.dk/, version 5.0_ALPHA) and TRANSFAC (http://www.gene-regulation.com/cgi-bin/pub/databases/transfac.cgi; version 7.0) public databases. We accept TFBS with a minimum relative score threshold of 0.8.

## Supplementary Information

Supplementary Information.
