# Author Correction: Regulatory rare variants of the dopaminergic gene *ANKK1* as potential risk factors for Parkinson’s disease

**DOI:** 10.1038/s41598-022-17301-0

**Published:** 2022-07-26

**Authors:** Estela Pérez‑Santamarina, Pedro García‑Ruiz, Dolores Martínez‑Rubio, Mario Ezquerra, Irene Pla‑Navarro, Jorge Puente, María José Martí, Francesc Palau, Janet Hoenicka

**Affiliations:** 1 Centro de Investigación Biomédica en Red de Enfermedades Raras (CIBERER), ISCIII, Madrid, Spain; 2 Centro de Investigación Príncipe Felipe (CIPF), Valencia, Spain; 3 Unit of Movement Disorders, Department of Neurology, Fundación Jimenez Díaz, Madrid, Spain; 4 Laboratory of Neurodegenerative Disorders, Department of Neurology, Hospital Clínic of Barcelona, IDIBAPS, Barcelona, Spain; 5 LabGenetics, Madrid, Spain; 6 Movement Disorders Unit, Department of Neurology, Hospital Clínic of Barcelona, IDIBAPS, Barcelona, Spain; 7 Laboratory of Neurogenetics and Molecular Medicine, Neurogenetics and Molecular Medicine Research Group, Institut de Recerca Sant Joan de Déu, C/ Santa Rosa 39‑57, Esplugues de Llobrega, 08950 Barcelona, Spain; 8 Department of Genetic Medicine, Hospital Sant Joan de Déu, Barcelona, Spain; 9 ICMID, Hospital Clínic, and Division of Pediatrics, University of Barcelona School of Medicine and Health Sciences, Barcelona, Spain; 10 Present Address: University of East Anglia, Norwich, UK; 11 Present Address: Unit of Rare Neurodegenerative Diseases, CIPF, Valencia, Spain; 12 Present Address: Rare Diseases Joint Units, CIPF-IIS La Fe and INCLIVA, Valencia, Spain

Correction to: *Scientific Reports* https://doi.org/10.1038/s41598-021-89300-6, published online 10 May 2021

The original version of this Article contained an error in the Acknowledgments section.

"This research was supported by the Fondo de Investigación Sanitaria, Instituto Salud Carlos III, Grant PI015/01013 and PI019/00126, Spain. The CIBERER is an initiative of the Instituto de Salud Carlos III. We acknowledge the ENCODE consortium and the ENCODE production laboratories that generated the data sets provided by the ENCODE Data Coordination Center that we used in this manuscript."

now reads:

"This study has been funded by Instituto de Salud Carlos III through the project "PI015/01013 and PI19/00126" Co-funded by European Regional Development Fund "A way to make Europe”. The CIBERER is an initiative of the Instituto de Salud Carlos III. We acknowledge the ENCODE consortium and the ENCODE production laboratories that generated the data sets provided by the ENCODE Data Coordination Center that we used in this manuscript."

The original Article has been corrected.

