# Supplementary material for: Regulatory rare variants of the dopaminergic gene ANKK1 as potential risk factors for Parkinson’s disease
Source: Sci Rep. 2021 May 10;11:9879. doi: 10.1038/s41598-021-89300-6 (PMC8110570; doi:10.1038/s41598-021-89300-6)
Supplement: Supplementary file 1 — Supplementary Information. [file 41598_2021_89300_MOESM1_ESM.pdf]

Supplementary file

Regulatory rare variants of the dopaminergic gene *ANKK1* as potential risk factors for Parkinson´s disease

Estela Pérez-Santamarina, Pedro García-Ruiz, Dolores Martínez-Rubio, Mario Ezquerra, Irene Pla-Navarro, Jorge Puente, María José Martí, Francesc Palau and Janet Hoenicka

Supplemental Methods:

Genetic panel

The genes included in the genetic panel were the following: *ANKK1* (Ankyrin repeat- and kinase domain-containing protein 1, MIM 608774), *ATP13A2* (ATPase 13A2, MIM 610513), *ATP6AP2* (ATPase, H+ transporting, lysosomal, accessory protein 2, MIM 300556), *ATP7B* (ATPase, Cu(2+)-transporting, beta polypeptide, MIM 606882), *COQ2*, (Parahydroxybenzoate-polyprenyltransferase, MIM 609825), *DNAJC13* (Dnaj/hsp40 homolog, subfamily c, member 13, MIM 614334), *DNAJC6* (Dnaj/hsp40 homolog, subfamily c, member 6, MIM 608375), *EIF4G1* (Eukaryotic translation initiation factor 4 gamma 1, MIM 600495), *FBXO7* (F-box protein 7, MIM 605648), *GBA* (Glucosidase beta acid, MIM 606463), *GCH1* (GTP cyclohydrolase I, MIM 600225), *HTRA2* (HTRA serine peptidase 2, MIM 606441), *LRRK2* (leucine-rich repeat kinase 2, MIM 609007), *NR4A2* (NM_006186.3), *PARK2* (Parkin, E3 ubiquitin protein ligase, MIM 602544), *PARK7* (Parkinsonism-associated deglycase, MIM 602533), *PINK1* (PTEN-induced putative kinase 1, MIM 608309), *PLA2G6* (Phospholipase A2 group VI, MIM 603604), *SLC6A3* (Dopamine transporter DAT1, MIM 126455), *SMPD1* (Sphingomyelin phosphodiesterase 1, MIM 607608, *SNCA* (alpha Synuclein, MIM 163890), *SYNJ1* (Synaptojanin 1, MIM 604297), *UCHL1* (Ubiquitin C-terminal hydrolase L1, MIM 191342) and *VPS35* (VPS35 retromer complex component, MIM 601501).

Expression vector constructions

Oligonucleotide primers containing engineered *Kpn*I sites (**Table S6**) were designed for PCR-based amplification of exon 1 and flanking regions of *ANKK1* gene (-327/+774 relative to the transcription start site) using Phusion High Fidelity DNA polymerase (Thermofisher, Waltham, Massachusetts, USA). The amplified products were cloned into the pCRII-TOPO vector (Invitrogen, Carlsbad, CA, USA). To generate the pGL3-ANKK1E1 plasmid for each variant, the recombinant pCRII-TOPO plasmids were digested with *Kpn*I to release and purify the insert

and finally inserted into *Kpn*I site in the luciferase reporter pGL3-basic vector (Promega, Fitchburg, Wisconsin, USA).

To generate the pGL3-ENH plasmids containing adenine (A) or timine (T) at rs7107223 position, oligonucleotide primers containing engineered *Kpn*I sites (**Supplementary Table 2**) were designed for PCR-based amplification. The amplified products were cloned into the pCR2.1-TOPO vector (Invitrogen), released from recombinant pCR1.2-TOPO plasmids by digestion with *Kpn*I, purified from gel and finally inserted into *Kpn*I site in the luciferase reporter pGL3-basic vector (Promega). The resulting constructs were sequenced to ensure sequence specificity.

To generate the control vector (T7-Ø), the *RELA* gene was excised from T7-RelA plasmid cutting with *Bam*HI and *Xba*I, and the resulting backbone was blunted using Mung Bean Nuclease (New England Biolabs) and religated.

Electrophoretic mobility shift assay (EMSA)

HEK293T (untreated and treated with apomorphine) and NHEK nuclear extracts were prepared using the NE-PER nuclear and cytoplasmic extraction reagents (Pierce). Double-stranded oligonucleotides (**Table S7**) were labeled with [γ-^{32}P] ATP using a T_4 polynucleotide kinase (Promega, Wisconsin, USA) and purified on a Microspin G-25 column (Roche, Basilea, Switzerland). For EMSA, 5 µg of nuclear extract were incubated with 1 µg of poly deoxyinosine-deoxycytosine and 15 fmol of the ^{32}P-labeled probes in a 20 µl binding reaction containing 1X buffer (50% glycerol; 10 mM Tris-HCl, pH 7.6; 500 mM KCl; 10 mM EDTA; and 1 mM dithiothreitol) for 30 min at room temperature. For the specificity control, a molar excess of unlabeled probe over the radiolabeled oligonucleotide was added to the binding reaction.

After, the reaction mixtures were mixed with loading dye and separated on a 6% nondenaturing polyacrylamide gel (29:1 acrylamide-bisacrylamide) and electrophoresed in a 0.25X Tris-boric buffer at 150 V for 1.5 h. The gel was dried and exposed to X-ray film for autoradiography.

RNA Extraction and RT-PCR form Peripheral Blood

A total of 2.5 ml whole venous blood was collected into PAXgene Blood RNA Tube (PAX tubes; BD Vacutainer, Plymouth, UK). Samples were stored for 2 hours at room temperature (RT), followed by immediate storage at -20°C until RNA extraction**.**

RNA was extracted using the PAXgene Blood RNA System Kit (PreAnalytiX, QIAGEN) following the manufacturer's guidelines. RNA yield and purity were assessed using NanoDrop 1000 Spectrophotometer (Thermo Scientific, Waltham, MA, USA), and RNA was stored at -80°C.

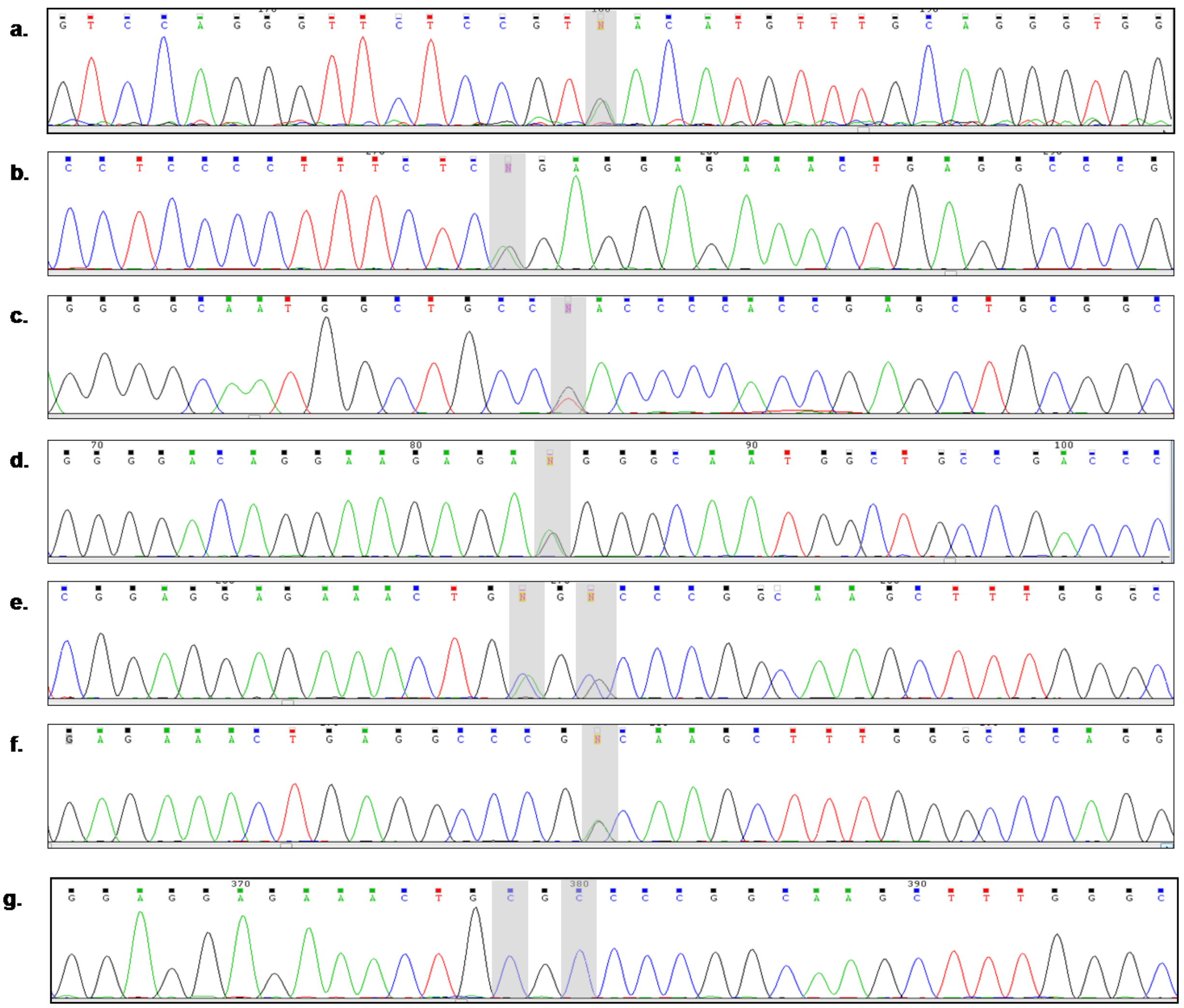

Figure S1. Sanger sequencing results of the *ANKK1* rare variants.

a. g.113384813A>G in heterozygosis, b. c.-6G>A in heterozygosis, c. c.10G>T (Asp4Tyr), d. c.185 +30 G>A in heterozygosis, e. c. [185+43A>C; 185+45G>C] in heterozygosis (in *cis*), f. c.185 +50 G>A in heterozygosis and g. c. [185+43A>C; 185+45G>C] in homozygosis (in *cis*), sequence from the TOPO 2.1 clone.

Homo sapiens	TTCTCGGAGGAGAAACTGAGGCCCGGCAAGC
Pan troglodytes	TTCTCGGAGGAGAAACTGAGGCCCGGCAAGC
Gorilla gorilla	TTCTCGGAGGAGAAACTGAGGCCCGGCAAGC
Pongo abelii	TTCTCGGAGGAGAAACTGAGGCCCGGCAAGC
Nomascus leucogenys	TTCTCGGAGGAAAAACTGAGGACCGGCAAGC
Macaca mulatta	TTCTGGGAGGAGAAACTGAAGCCCGGCAAGC
Macaca fascicularis	TTCTCGGAGGAGAAACTGAAGCCCGGCAAGC
Papio anubis	TTCTCGGAGGAGAAACTGAGGCCCGGCAAGC
Chlorocebus sabaeus	TTCTCGGAGGAGAAACTGAGGCCCGGCAAGC
Callithrix jacchus	TTCTCGGAGGAGAAACTGAGGCCGGGCAAGC
Saimiri boliviensis	TTCTCGGAGAAGAAACTGAGGCCGGGCAAGC

Figure S2. Comparison of the first intron of *ANKK1* gene nucleotide sequence among primates.

Grey boxes show the position where the *ANKK1* rare intronic variants are located. c.185 +30 G>A, c.[185+43A>C; 185+45G>C] and c.185 +50 G>A occur in well-conserved nucleotides among primates.

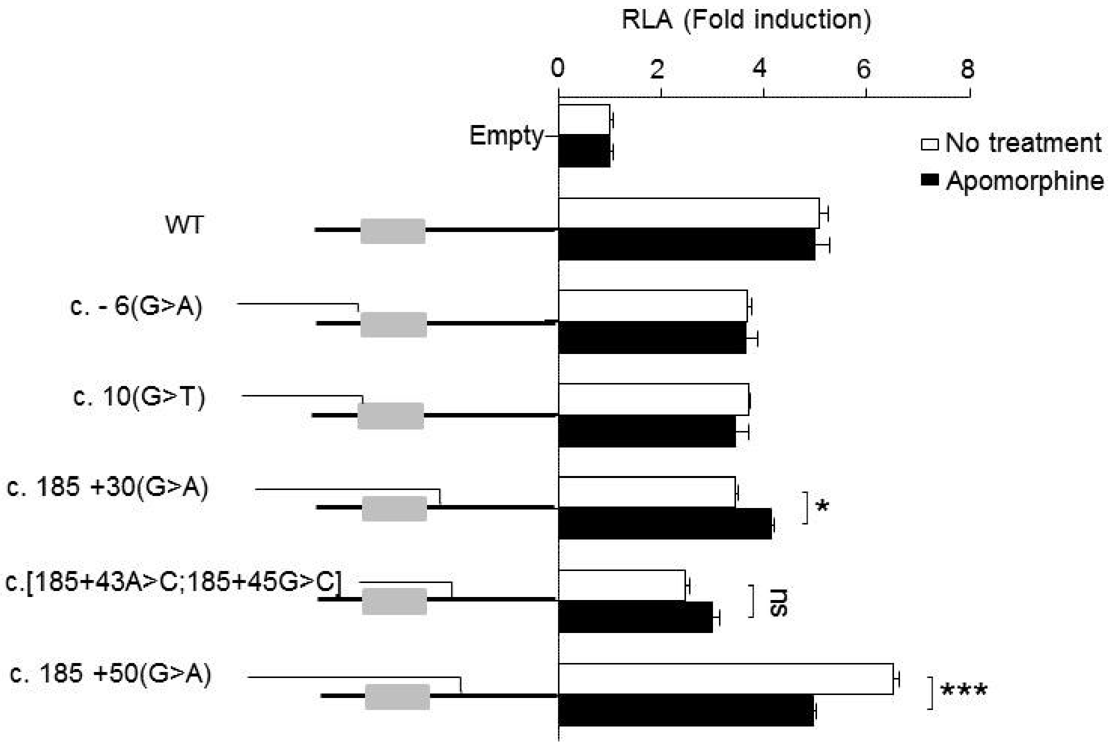

Figure S3. Apomorphine treatment effect upon PD-related *ANKK1* rare SNVs.

Relative luciferase activity (RLA) of WT and variants of *ANKK1* exon 1/intron 1 (sequence -327 up to +774) cloned in the pGL3-basic vector and transfected in HEK 293T cells. Data are shown as mean ±SEM (N=4). The assay was carried out in HEK293T (untreated and treated with apomorphine). Asterisks indicated statistical significance (*p<0.05, **p<0.01, ***p<0.001), one-way ANOVA followed by Tukey correction was conducted.

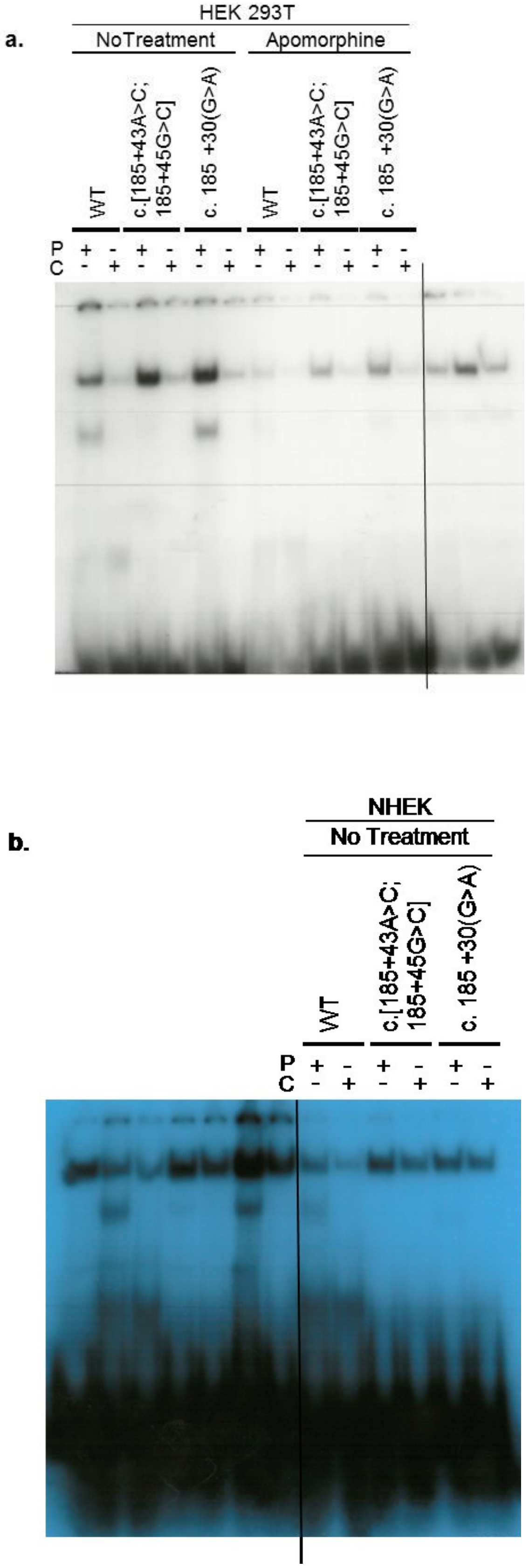

Figure S4. Full-length EMSA gels for Figure 2

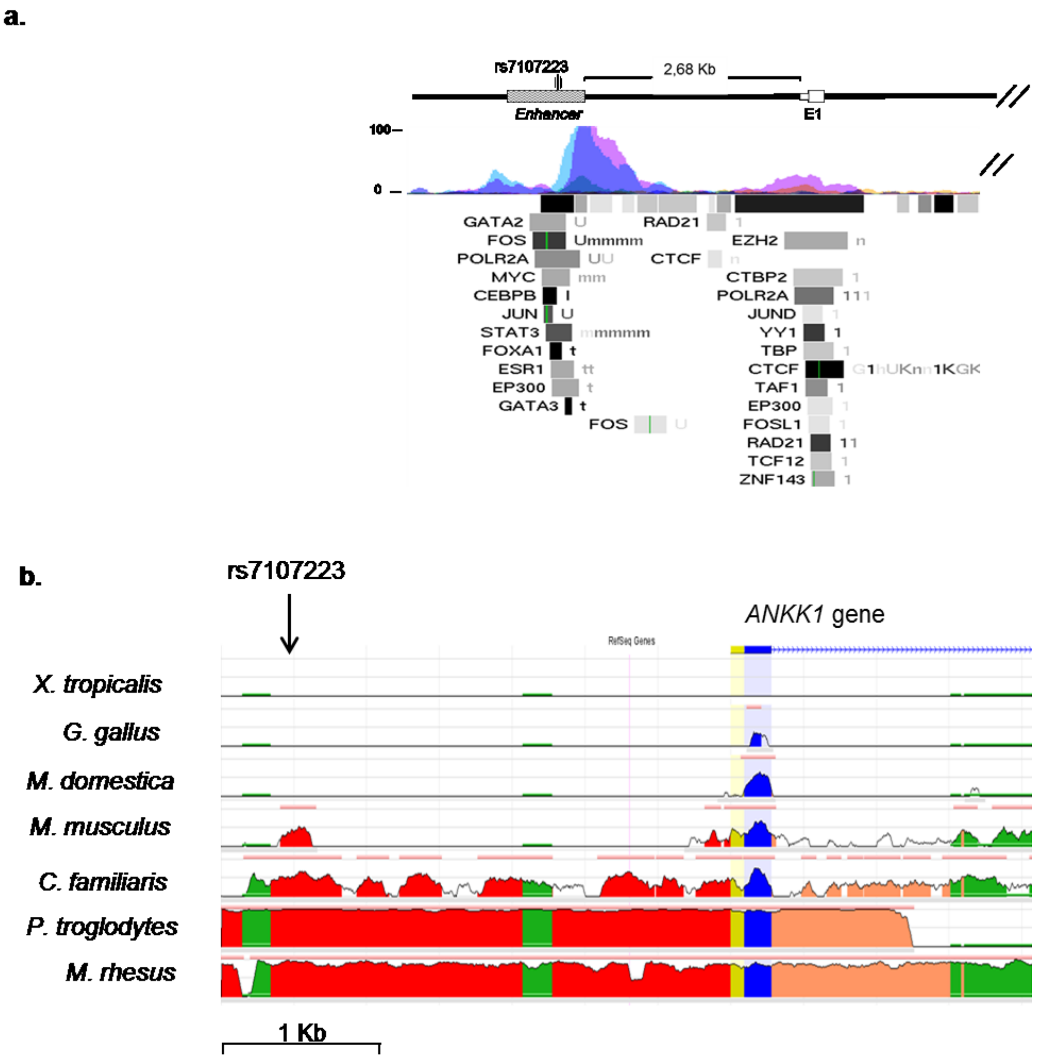

Figure S5. *ANKK1* upstream regulatory region in silico studies

A. ENCODE epigenetics marks of *ANKK1* enhancer and promoter including histone H3 lysine 27 acetylation (H3K27Ac), histone H3 lysine 3me1 (H3K4me1), DNase I hypersensitive site and transcription factors binding sites in Human embryonic stem cells (promoter), Human Skeletal Muscle Myoblasts (Enhancer), Human Umbilical Vein Endothelial Cells (Enhancer), Normal human epidermal keratinocytes (Enhancer and promoter) cell lines. **B.** ECR Browser visualization of the *ANKK1* gene in the genome (http://ecrbrowser.dcode.org/). The image shows the conservation profiles of the human region in comparison with the chimpanzee (*P. troglodytes*), dog (*C. familiaris*), cow (*B. Taurus*), mouse (*M. musculus*), opossum (*M. domestica*), chicken (*G. gallus*) and zebrafish (*D. rerio*) genomes. Minimum conservation of 50% is displayed. A conserved alignment is blue if it overlaps with a coding exon, UTR's regions are in yellow, introns in salmon, the intergenic regions in red, and the color green indicates repetitive elements in the base sequence.

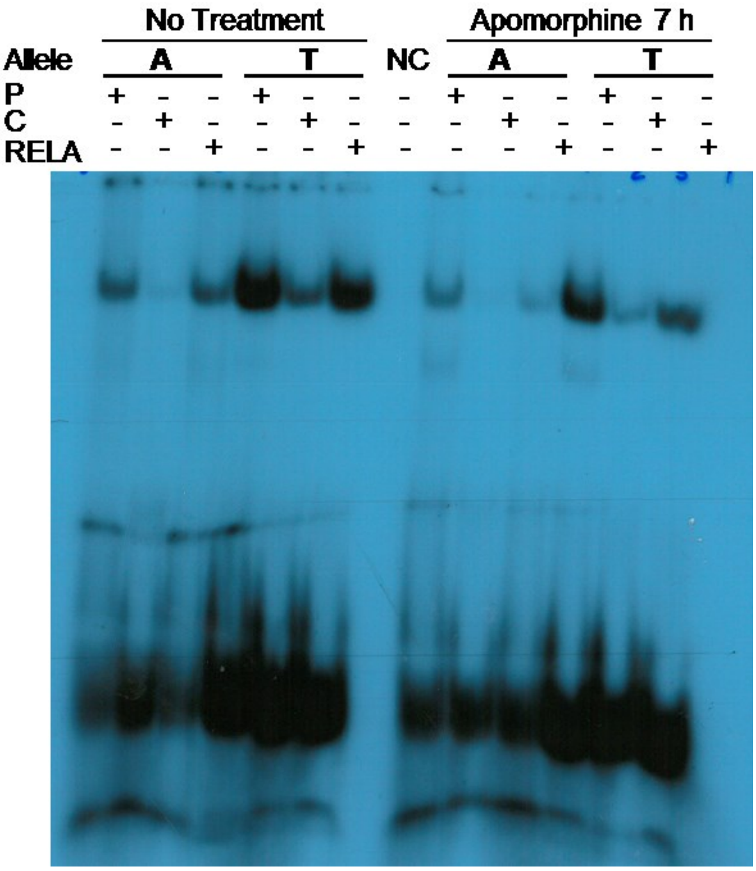

Figure S6. Full-length EMSA gels for Figure 3

Table S1. Predicted effects and conservation scores of *ANKK1* SNVs found in patients with PD

			PREDICTED EFFECT		CONSERVATION SCORE		
POSITION	SNV	DATA BASE FREQUENCY GNOMAD/ CSVS	CADD[a]	MUTTASTER2[b]	PHYLOP[c]	PHASTCONS[d]	GERP++[e]
5´UTR Chr11: 113258601	c.-6G>A	NR	1.509	P	-2.086	0	-4.29
Exon 1 Chr11: 113258616	c.10G>T rs3567708	0.001817/ 0.00213	10.82	P	-0.163	0	-1.3
Intron 1 Chr11: 113258821	c.185+30G>A rs545702309	0.0001568/ 0.000634	5.908	P	-0.501	0	-3.6
Intron 1 Chr11: 113258834	c.185+43A>C rs769430211	0.0001548/ NR	9.452	P	0.644	0.173	1.98
Intron 1 Chr11: 113258836	c.185+45G>C rs780054479	0.0003005/ NR	11.04	DC	2.162	0.243	3.35
Intron 1 Chr11: 113258841	c.185+50G>A rs1469219226	0.00001571/ 0	3.79	P	-0.267	0	-1.19

Abbreviations: P, polymorphism; DC, Disease-causing; NR, not reported.
CSVS - Collaborative Spanish Variant Server https://www.ciberer.es/en/platforms/bier
GnomAD - genome aggregation database http://gnomad.broadinstitute.org/
a CADD [1]
b MutTaster [2]
c PhyloP scores range between –20 and 7.532.
d PhastCons scores range between 0 and 1 [3]
e GERP scores range between –12.36 and 6.18 [4]
f Nucleotide numbering uses +1 as the A of the ATG translation initiation codon in the reference sequence NM_178510.1

Table S2. Prediction of gain or loss of transcription factors binding sites associated with rare SNVs at the *ANKK1* locus

SNV	TF LOST	TF GAIN
c.-6G>A	SP2, SP1, Klf4, CEBPA, MZF1, CEBPB	PRDM1, Gata1, Esrrb, GTA3, SOX10, NR4A2
c.10G>T	E2F1, EGR2	SP1, FOXC1
c.185+30G>A	–	Gata4, EBF1, STAT5
c.[185+43A>C;185+45G>C]	STAT2, Rxra, Zfx, E2F1	AR, INSM1, NRF1, KLF4 RFX5
c.185+50G>A	E2F1, Zfp423	TFAP2C

TF: transcription factor

Table S3. NGS panel results of PD patients carrying *ANKK1* rare variants

**PATIENT**	***ANKK1* SNV**	**NGS findings (nucleotide/protein) CSVS frequency**	**NGS findings classification: ACMG-AMP [5]** (accessed on 21th, February 2021) **and HGMD ***
PD-01	c.[185+43A>C;185+45G>C]	***NR4A2***<br>c.580T>C , p.Phe194Leu<br>CSVS: 0	VUS<br>Nf
PD-02 (DNA Bank)	c.185 +30 G>A	-	-
PD-03	c.10G>T, p.Asp4Tyr	None	
PD-04	c.-6G>A	***ATP7B***<br>c.1182G>C, p.Leu394Phe<br>rs201874048 CSVS: 0	VUS<br>Nf
PD-05	c.185+50 G>A	None	
PD-06	c.[185+43A>C;185+45G>C]	***ANKK1***<br>c.850G>A, p.Glu284Lys<br>rs45466992 CSVS: 0	LB<br>Nf
PD-07	c.10G>T, p.Asp4Tyr	***ATP7B***<br>c.4135C>T, p.Pro1379Ser<br>rs181250704 CSVS:0.007	VUS<br>DM
PD-08	c.10G>T, p.Asp4Tyr	***GBA***<br>c.115+1G>A, IVS3+1G>A<br>CSVS: 0.005<br><br>***ATP7B***<br>c.4135C>T, p.Pro1379Ser<br>CSVS:0.007	P<br>DM(Gaucher disease)<br><br>VUS<br>DM
PD-09	g.113384813A>G	***LRRK2***<br>*c.*382A>G, p.Ser128Gly<br>rs187299177 CSVS*:* 0.001	LB<br>Nf
PD-10	g.113384813A>G	***ATP7B***<br>c.3688A>G, p.Ile1230Val<br>rs200911496 CSVS: 0<br><br>***LRRK2***<br>c.6566A>G, p.Tyr2189Cys<br>rs35658131 CSVS: 0.002	LP<br>DM<br><br>LB<br>DM (Parkinson's disease)

CSVS - Collaborative Spanish Variant Server https://www.ciberer.es/en/platforms/bier
ACMG-AMP: American College of Medical Genetics and Genomics and the Association for Molecular Pathology. VUS: variant of uncertain significance, LB: likely benign, P: pathogenic, LP: likely pathogenic.
HGMD: Human Genome Mutation Database, Nf: Not found, DM: Disease mutation.

Table S4. Clinical data of PD patients carrying *ANKK1* rare variants

Patient	Age at onset	Main Symptoms (B R T)	Motor complications (F D GF)	Cognitive decline	Response to Medication
PD-01	53	B T	F D F	1	1
PD-03	71	B	F	0	1
PD-04	68	B T	No	0	No
PD-05	76	B R T	F D G F	1	1
PD-06	79	-	G F	1	-
PD-07	45	-	-	0	-
PD-08	57	B R	F D	1	?
PD-09	60	B T	F D	1	?
PD-10	57	B T	F D G F	1	?

Main symptoms: **B** Bradykinesia, **R** Rigidity, **T** Tremor.
Motor complications: **F** Fluctuations, **D** Dyskinesia, **GF** Gait Freezing.
1: presence; **0**: absence.

Table S5. Oligonucleotide and amplicon information of *ANKK1* exons and 5'UTR enhancer.

ANKK1 region	Primer pairs	Oligonucleotide sequence (5'- 3')	Size (pb)	AT (ºC)	Chromosomal Location (hg38)
Exon 1	ANKK1E1–D<br>ANKK1E1–R	ACCCGAGGAGCAGGAAGCG<br>TCCTCAGCCCCAAACTCAGC	384	65	11:113387794<br>11:113388177
Exon 2	ANKK1E2–D<br>ANKK1E2–R	TTCCACTACCTTGCAAGCTCC<br>TGGATAATTCTGCTCACCTGG	499	65	11:113393346<br>11:113393844
Exon 3	ANKK1E3–D<br>ANKK1E3–R	GAACAGGCAGATAGCAGGAGG<br>AGACCTCATGCCCGCACTGC	354	60	11:113394835<br>11:113395188
Exon 4	ANKK1E4–D<br>ANKK1E4–R	TCAACTAAGTCATTCAGAAAGG<br>CCTTTCCTTTCCTTTAGC	296	56	11:113395320<br>11:113395615
Exon 5	ANKK1E5–D<br>ANKK1E5–R	CTCCAGCCCTACCTCTC<br>GTCCTTCTAGGCCGTAGTACC AGC	325	68	11:113396018<br>11:113396342
Exon 6	ANKK1E6–D<br>ANKK1E6–R	ATGGATTTTGGGAGACAGG<br>CTCCAGCTTAGCATGACC	267	56	11:113397122<br>11:113397388
Exon 7	ANKK1E7–D<br>ANKK1E7–R	ACTGATCTCCACCCTGCCTGC<br>ACTTGAAAGGGAGGCAGCCAGG	277	68	11:113397942<br>11:113398218
Exon 8a	ANKK1E8a–D<br>ANKK1E8a–R	AGGGGGATGGCCATGATGACC<br>CAAAGTAGGCGGCCACATGGAGG	590	60	11:113398893<br>11:113399504
Exon 8b	ANKK1E8b–D<br>ANKK1E8b–R	CAGAATAACTTTGAGAATGTGG<br>GTTGATGACACTCAGGAAGG	624	60	11:113399860<br>11:113391000
Exon 8c	ANKK1E8c–D<br>ANKK1E8c–R	CACCTAGCTGCACGCCACGG<br>TGCCTCAGCCTCCCAAAGTGC	469	65	11:113399860<br>11:113400328
Enhancer 5´UTR	ENHsec–D<br>ENHsec–R	CTAGTATATACCTAAGGAATGTAACC<br>CAGATTGAAGATGGAAGTGTTC	796	65-55*	11:113384266<br>11:113385061

AT: annealing temperature. * Touch down PCR

Table S6. Primers used for cloning, sequencing and RT-PCR procedures.

Primer pairs	Oligonucleotide sequence (5'- 3')	Size (pb)	AT (ºC)	Chromosomal Location (hg38)
rs7107223-F<br>rs7107223-R	AAGAAGCCAGGGTTTAAATGC<br>TGTGATTGTCTTTGGGGAACC	403	69-62*	11:113384600<br>11:113385003
KpnI+E1ANKK1-F<br>KpnI+E1ANKK1-R	ttcttaGGTACCGACACCTTCTCCCAGCATCC<br>tacataGGTACCGAGAGAACTCCAGACTTGACC	1126	63	11:113388659<br>11:113387558
SalI+ENH-F<br>SalI+ENH-R	taaataGTCGACCAGCTGGTTCATATTTTTTGCC<br>ttattaGTCGACACCCCAAAATGCTCTGAGTCC	909	65	11:113384233<br>11:113385117
KpnI+ENH-F<br>KpnI+ENH-R	taaataGGTACCCAGCTGGTTCATATTTTTTGCC<br>ttattaGGTACCACCCCAAAATGCTCTGAGTCC	909	65	11:113384233<br>11:113385117
PPIA-F<br>PPIA-R	GACTGAGTGGTTGGATGGCA<br>TGGTCTTGCCATTCCTGGAC	104	57	8:44801371<br>8:44801371
GAPDH-F<br>GAPDH-R	CGGAGTCAACGGATTTGGTC<br>AATCATATTGGAACATGTAAACCATGTAGT	134	57	13:6534849<br>13:6536692
ANKK1FL-F<br>ANKK1FL-R	CCCAAGAAGAGGCCATGCTT<br>GTCCTCATTAACCTCCCCGG	156	57	11:113396198<br>11:113397991

AT: annealing temperature * Touch down PCR

Table S7. Oligomers used in Electrophoretic Mobility Shift Assays (EMSA)

EMSA Oligomer	Oligonucleotide sequence (5’- 3’)	Chromosomal location (hg38)
NFkB-D<br>NFkB-Rev	AGTTGAGGGGACTTTCCCAGGC<br>GCCTGGGAAAGTCCCCTCAACT	*
rs107223 A-D<br>rs7107223 A-Rev	GGGCAGACTGGGAAACTTCCCTATATGCAA<br>TTGCATATAGGGAAGTTTCCCAGTCTGCCC	11:113384838
rs7107223 T-D<br>rs7107223 T-Rev	GGGCAGACTGGGAATCTTCCCTATATGCAA<br>TTGCATATAGGGAAGATTCCCAGTCTGCCC	11:113384838
Intron1 wt-D<br>Intron1 wt-Rev	TTCTCGGAGGAGAAACTGAGGCCCGGCAAGC<br>GCTTGCCGGGCCTCAGTTTCTCCTCCGAGAA	11:113388094
Intron1 c.185+43y+45-D<br>Intron1 c.185+43y+45-Rev	TTCTCGGAGGAGAAACTGCGCCCCGGCAAGC<br>GCTTGCCGGGGCGCAGTTTCTCCTCCGAGAA	11:113388094
Intron1 c.185+30-D<br>Intron 1c.185+30-Rev	TTCTCAGAGGAGAAACTGAGGCCCGGCAAGC<br>GCTTGCCGGGCCTCAGTTTCTCCTCTGAGAA	11:113388094

*Reference [6]

REFERENCES

1 Kircher, M. *et al.* A general framework for estimating the relative pathogenicity of human genetic variants. *Nat Genet* **46**, 310-315 (2014).

2 Schwarz, J. M., Cooper, D. N., Schuelke, M. & Seelow, D. MutationTaster2: mutation prediction for the deep-sequencing age. *Nat Methods* **11**, 361-362 (2014).

3 Pollard, K. S., Hubisz, M. J., Rosenbloom, K. R. & Siepel, A. Detection of nonneutral substitution rates on mammalian phylogenies. *Genome Res* **20**, 110-121 (2010).

4 Davydov, E. V. *et al.* Identifying a high fraction of the human genome to be under selective constraint using GERP++. *PLoS Comput Biol* **6**, e1001025 (2010).

5 Richards, S. *et al.* Standards and guidelines for the interpretation of sequence variants: a joint consensus recommendation of the American College of Medical Genetics and Genomics and the Association for Molecular Pathology. *Genet Med* **17**, 405-424 (2015).

6 Lu, T. *et al.* Secreted transforming growth factor beta2 activates NF-kappaB, blocks apoptosis, and is essential for the survival of some tumor cells. *Proceedings of the National Academy of Sciences of the United States of America* **101**, 7112-7117 (2004).
